# Child-Welfare Progress

**Published:** 1920-04-03

**Authors:** 


### Child-Welfare Progress.

It is well to find that the Minister of Health gives credit to the child-welfare centres for their share in the improved infant statistics which were published last week. Speaking at a cripple mission exhibition in Hoxton, Dr. Addison said one of the chief aims of the Ministry during its first year of existence was to increase by financial aid of a very generous kind the utility of all those services which were concerned with child life. Many hundreds of centres had been established, and he was glad to see the great improvement in the infant death-rate. There seems no doubt the greatly increased efforts for child welfare in all directions are now having demonstrable effect.

In an earlier statement made in Parliament, Dr. Addison mentioned that during the year 400 additional child welfare centres were established throughout the country, and that the infant mortality rate of last year, 89 per 1,000, was the lowest on record. In 1906, before the maternity work had been commenced, the rate of infant mortality was 145; while in 1914, when various centres had been formed, the rate had gone down to 105 per 1,000. These facts should give great encouragement to all workers in this movement. When so great a number of centres have been established, with so many health visitors engaged under the various medical officers of health, and so many facilities provided for mothers and children obtaining medical and other attentions, that work must show fruit.

